# Intravenous and subsequent long-term oral tranexamic acid in enhanced-recovery primary total knee arthroplasty without the application of a tourniquet: a randomized placebo-controlled trial

**DOI:** 10.1186/s12891-019-2885-5

**Published:** 2019-10-25

**Authors:** Hao-Yang Wang, Liu Wang, Ze-Yu Luo, Duan Wang, Xin Tang, Zong-Ke Zhou, Fu-Xing Pei

**Affiliations:** 1 Department of Orthopedics, West China Hospital/West China School of Medicine, Sichuan University, 37# Wuhou Guoxue road, Chengdu, 610041 People’s Republic of China; 2 State Key Laboratory of Oral Diseases & National Clinical Research Center for Oral Diseases, Sichuan University, Chengdu, 610041 People’s Republic of China; 3 Department of Cariology and Endodontics, West China Hospital of Stomatology, Sichuan University, Chengdu, 610041 People’s Republic of China

**Keywords:** Total knee arthroplasty, Tranexamic acid, Blood loss, Swelling

## Abstract

**Background:**

To assess the efficacy and safety of intravenous and subsequent long-term oral tranexamic acid (TXA) following total knee arthroplasty (TKA) without a tourniquet.

**Methods:**

In this double-blinded trial, 118 patients undergoing primary TKA were randomized into two groups: the patients in group A received intravenous TXA at 20-mg/kg 10 min before the surgery and 3 h postoperatively, and then oral 1 g TXA from postoperative day (POD) 1 to POD 14, and the patients in group B received intravenous TXA at 20-mg/kg 10 min before surgery and 3 h postoperatively, and then oral 1 g placebo from postoperative day (POD) 1 to POD 14. The primary outcome was total blood loss. Secondary outcomes included ecchymosis area and morbidity, postoperative transfusion, postoperative laboratory values, postoperative knee function and length of hospital stay. Complications, and patient satisfaction were also recorded.

**Results:**

The mean total blood loss was lower in Group A than in Group B (671.7 ml vs 915.8 ml, *P* = 0.001). There was no significant difference in the transfusion rate between the two groups. Group A had a higher hemoglobin than Group B on POD 3 (106.0 g/L vs 99.7 g/L, *P* = 0.001). However, no significant difference was found for Hb or hematocrit on POD 1 or POD 14 between the two groups. Patients in Group A had less ecchymosis morbidity (7 vs 38, *P* = 0.001), smaller ecchymosis area (1.6 vs 3.0, *P* = 0.001) than Group B. The blood coagulation level as measured by fibrinolysis (D-Dimer) was lower in Group A than in Group B on POD 1 and POD 3 (4.6 mg/L vs. 8.4 mg/L, respectively, *P* = 0.001; 1.5 mg/L vs. 3.3 mg/L, respectively, *P* = 0.001). However, there was no significant difference on POD 14, and the fibrin degradation products showed the same trend. Patients in Group A had less swelling than those in Group B on POD 3 and POD 14. The circumference of the knee was 43.1 cm vs. 46.1 cm (POD 3, *P* = 0.001) and 41.4 cm vs. 44.9 cm (POD 14, *P* = 0.001) in Group A vs Group B, respectively. Nevertheless, the circumference of the knee in the two groups was similar on POD 1 and POD 3 M. No significant differences were identified in knee function, pain score, or hospital stay. No significant differences were identified in thromboembolic complications, infection, hematoma, wound healing and patients satisfaction between the two groups.

**Conclusion:**

Intravenous and subsequent long-term oral TXA produced less blood loss and less swelling and ecchymosis compared with short-term TXA without increasing the risk of complications.

**Trial registration:**

The trial was registered in the Chinese Clinical Trial Registry (ChiCTR-IPR-17012264).

## Background

Total knee arthroplasty (TKA) is viewed as one of the most successful orthopedic surgeries for most end-stage knee diseases [1]. In recent years, the concept of enhanced recovery after surgery (ERAS) has greatly improved the speed of rehabilitation after total joint arthroplasty. Patients feel more comfortable during the perioperative period and have shorter hospital stays and lower cost [2, 3]. Tranexamic acid (TXA) plays an important role in ERAS due to its antifibrinolytic function [4]. TXA can prevent the activation of plasminogen and delay fibrinolysis and reduce blood loss and the rate of blood transfusion perioperatively [5–7]. Recent studies showed that TXA may be administered intravenously, intra-articularly, or orally [8–11].

Tourniquets can reduce blood loss during operations and provide a better view for surgeons, but the use of a tourniquet may be related to muscle damage, paralysis, severe thigh pain, delayed rehabilitation, and reduced patient satisfaction [12, 13]. Considering these concerns, not using a tourniquet during TKA surgery can avoid those side effects and promote recovery postoperatively. Similarly, some studies of tourniquet use during TKA showed that avoiding tourniquets can reduce pain and edema from the operation side of the lower limb without increasing blood loss with use of multiple doses of TXA [14, 15].

Many guidelines recommend that anticoagulants be used during TKA to prevent venous thromboembolism (VTE), including deep vein thrombosis (DVT) and pulmonary embolism (PE) after TKA [16, 17]. New oral anticoagulants, such as Rivaroxaban and Apixaban, can effectively reduce the occurrence of DVT and PE, but there are still some side effects when patients take these drugs, including swelling, ecchymosis, or wound complications. These side effects may make patients panic and are disadvantageous to recovery after surgery [18–22].

A large number of studies confirmed that intravenous, intra-articular, or oral use of tranexamic acid can reduce blood loss and be helpful for reducing inflammation and swelling from limb surgery, reducing the occurrence of subcutaneous ecchymosis, but does not increase the incidence of thrombosis [6, 9, 14, 23–26]. In our clinical work, we found that during the anticoagulation process (from the day of surgery to POD 14) after joint replacement, most patients may have various degrees of subcutaneous ecchymosis. We considered that subcutaneous ecchymosis may be caused by anticoagulant drugs that can induce hidden blood loss or subcutaneous capillary bleeding. Subcutaneous ecchymosis was one of the main problems indicated by patients during the follow-up visit and was not conducive to early postoperative rehabilitation. Studies have supported that the use of tranexamic acid during hospitalization could reduce the incidence of subcutaneous ecchymosis in patients undergoing arthroplasty, and we wondered whether prolonged use of tranexamic acid postoperatively could enhance patient recovery and reduce the incidence of subcutaneous ecchymosis after discharge. However, in almost all of these previous studies, the use of TXA occurred in the hospital, and there has been no related research about the efficacy and safety of long-term use of oral TXA after TKA. Therefore, we designed a prospective, randomized, double-blind placebo trial conducted in an enhanced recovery setup at our institution to assess the efficacy and safety of extended oral tranexamic acid after primary unilateral total knee arthroplasty with no tourniquet or drain. We hypothesized that, compared with placebo, after total knee arthroplasty, extended oral TXA would reduce blood loss, incidence of subcutaneous ecchymosis and limb swelling with Rivaroxaban to prevent thrombosis post-discharge without increased thromboembolic complications.

## Methods

### Patients

This study was conducted at the Department of Joint Surgery in West China Hospital, Sichuan University, and registered in the Chinese Clinical Trial Registry (ChiCTR-IPR-17012264). The Institutional Review Board (Medical Ethics Committee of West China Hospital, Sichuan University) give permission for our study.

From September 2017 and April 2018, patients with primary osteoarthritis undergoing primary unilateral TKA were screened for enrollment. Perioperative management of surgery was based on a widely recognized multimodal enhanced recovery strategy, including VTE prevention, pain control [27–29], blood loss management [30], and rehabilitation training [31, 32]. The exclusion criteria were secondary osteoarthritis, allergy to this medicine, a history of coexisting diseases that cannot tolerate surgery or general anesthesia, and active cancer.

### Drug delivery and randomization

All patients were randomized into two groups (Group A: IV and subsequent oral TXA; Group B: IV TXA only) based on a computer-generated randomization list generated using Randomization.com. The randomization was prepared by a statistician who was not involved in this clinical trial. The randomization assignments were placed into sequentially numbered opaque sealed envelopes, which were kept by a certificated research pharmacist. The envelope was opened on the day of operation, and the corresponding drug and placebo were handled by a researcher who was not involved in patient care. The patients, trial participants, anesthesiologists, outcome assessors, and data collectors were blinded to allocation. The placebo (starch tablet) that had the same appearance as oral TXA was provided from a specific department in our hospital. The patients in Group A were given TXA (20 mg/kg) intravenously 10 min before the surgery and 3 h after the operation, and then the patients received 1 g TXA orally from postoperative day (POD) 1 to POD 14. Patients in the Group B received IV TXA (20 mg/kg) intravenously 10 min before the surgery and 3 h after the operation, and then the patients received placebo pills identical in quantity to oral TXA from POD 1 to POD 14. All the operations were performed by a senior doctor.

### Surgical procedure and postoperative management

All patients were under general anesthesia and used a medial para-patellar approach. All patients also received standard analgesia perioperatively [27, 28], including adductor canal block (30 ml 0.33% ropivacaine) before the operation and periarticular multisite infiltration (40 ml 0.25% ropivacaine) before fixing the prosthesis. A tourniquet, intraarticular drainage tube, and pressure dressing were not used in any patients in this study [22, 33]. All of the patients had a doppler ultrasound conducted by experienced ultrasound doctor to diagnose DVT before the operation, at POD 14 and 3 months postoperatively. PEs were detected by clinical symptoms and contrast-enhanced chest CT scans if it’s necessary.

During hospitalization, all patients received low molecular weight heparin (Clexane, Sanofi-Aventis, France) and a venous pump (the first night after the operation) to prevent VTE. Patients were asked to perform equal length contractions of the femoral quadriceps and ankle pump movements 20 times per minute as soon as they recovered from anesthesia. Rivaroxaban (10 mg, Xarelto, Bayer, Germany) was administered orally once a day for another 10 days after determining no bleeding events occurred.

Based on the guidelines of the Chinese Ministry of Health, an allogeneic transfusion was given if Hb was < 70 g/L in asymptomatic patients or between 70 g/L and 100 g/L in symptomatic patients (i.e. fatigue, poor appetite, anemia, or myocardial ischemia) in hospital. An attending physician who did not participate in the study made the transfusion decision [34].

#### Outcome measurements

Patient’s age, sex, American Society of Anesthesiologists (ASA) classification, Caprini score, weight, height, body mass index (BMI) and predicted blood volume (PBV) were collected for comparison. The primary outcomes was total blood loss (TBL), and estimated blood loss was calculated applying the Gross formula [35]:

$$ \mathrm{Total}\ \mathrm{blood}\ \mathrm{loss}=\mathrm{PBV}\ \mathrm{x}\ \left(\mathrm{Hct}\ \mathrm{pre}-\mathrm{Hct}\ \mathrm{post}\right)/\mathrm{Hct}\ \mathrm{ave} $$

- PBV = patient’s blood volume
- Hct pre = the initial preoperative hematocrit level
- Hct post = the lowest postoperative hematocrit level during hospitalization or the lowest postoperative hematocrit prior to blood transfusion
- Hct ave = the average of Hct pre and Hct post

The PBV was assessed according to the formula from Nadler et al [36]:

PBV (mL) = k1 x height (m) + k2 x weight (kg) + k3; k1 = 0:3669, k2 = 0:03219, and k3 = 0:6041 for men; k1 = 0:3561, k2 = 0:03308, and k3 = 0:1833 for women. If a reinfusion or an allogenic transfusion was conducted, the volume transfused was added when calculating TBL.

The secondary outcomes included the subcutaneous ecchymosis morbidity, area of ecchymosis, patients requiring transfusion, units transfused, and postoperative laboratory values (i.e. hemoglobin, hematocrit, FDP, and D-Dimer). Postoperative knee function, knee circumference, VAS pain score, and length of hospital stay were also assessed as the secondary outcomes. Hemoglobin was measured before the operation and at POD 1, 3, and 14. Based on previous literature and our own experience, the patient’s hemoglobin level was minimized on the third day after TKA. We chose POD 3 to measure total blood loss. Two experienced clinicians who did not participate in the study judged the degrees of ecchymosis and swelling, which were consistent with our previous studies [33]. If subcutaneous ecchymosis was found at any part of the surgical limb, the range of ecchymosis was measured as the percentage of body surface area (area of the patient’s own palm defined as approximately 1% of body surface area, referenced in the estimation of burn area by Lund-Browder [37]. Knee circumference was measured as thigh circumference 10-cm proximal to the patellar. If the surgical side was over than the contralateral limb was defined as positive. Limb swelling was measured before operation and on POD 1, 3, and 14 and after 3 months. As for complications, we recorded complications (DVT, infection, and wound secretion), 30-day mortality, and 90-day readmission. Marsh’s Satisfaction Questionnaire was used to investigate patient satisfaction.

#### Sample-size calculations

The sample size was calculated on the basis of the difference in the primary outcome, namely, total blood loss. Based on pilot data from 66 patients who received unilateral primary TKA with use of the same fast-track program between March 2016 and November 2016, the mean total blood loss was 978 ml (SD 229). The relevant additional clinical effect of reduced total blood loss based on the added use of oral TXA following its use preoperatively was found to be 164 ml in the historical cohort [38] . Based on these parameters, it was determined that 48 patients were required per group, with an assumed alpha of 5% and a power of 95%. To allow for loss to follow-up of 20%, 60 patients were needed for each group. Calculations were performed with G*Power 3.1.

### Statistical analysis

The mean and standard deviation (SD) were calculated for the quantitative data, and frequencies and percentages were calculated for qualitative data. Before any analyses were performed, the distributions of all variables were tested by the Kolmogorov–Smirnov test. An unpaired Student’s t-test was used when the data appear to be normally distributed. Otherwise, the Mann–Whitney U test was used. The chi-squared or Fisher’s exact tests was performed to analyze categorical variables. Significance was set at *p* < 0.05. Analyses were performed using SPSS Version 22.0 (IBM Corp., Armonk, New York).

## Results

### Patients

**Fig. 1:**
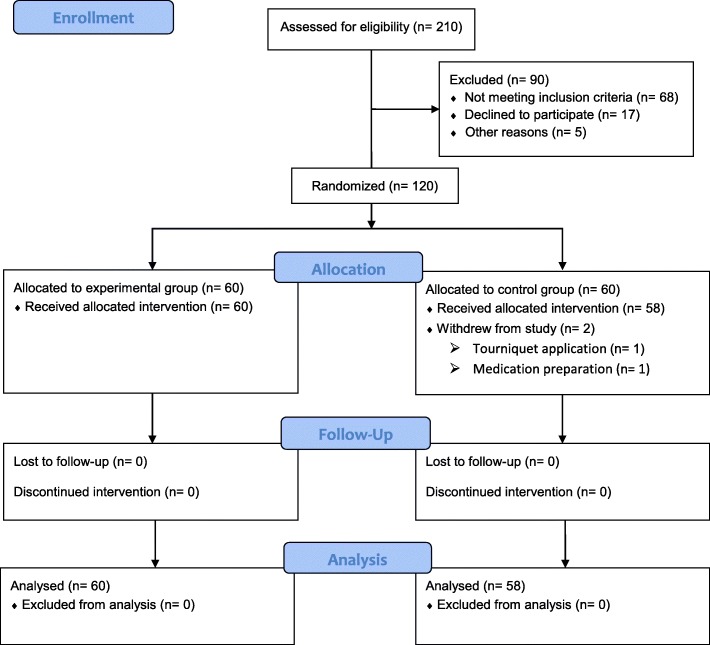
CONSORT (Consolidated Standards of Reporting Trials) flow diagram

From September 2017 to April 2018, 210 patients scheduled for primary unilateral TKA were screened for participation in our trial. Ninety patients were excluded, and the remaining 120 patients underwent randomization into two groups: Group A (IV and subsequent oral TXA group, *n* = 60) and Group B (IV TXA group, *n* = 60). Two patients in Group B were excluded because of tourniquet application (1 patient) and medication not being prepared in time (1 patient) (Fig. 1). No patient was lost or excluded during follow-up. No significant differences between the two groups were identified with respect to demographic data, operative time, Caprini score, knee function, or preoperative laboratory values (Table 1). The follow-up duration was 3 months after surgery.

**Table 1:** Baseline characteristics and perioperative demographics

Variable	Group A (*N* = 60)	Group B (*N* = 58)	*P* value
Patient characteristics			
Age (year)^b^	63.0 ± 13.9	64.1 ± 9.3	0.62
Gender (male/female) ^a^	15/45	11/47	0.43
Height (m)^b^	1.5 ± 0.06	1.6 ± 0.07	0.07
Weight (kg)^b^	60.9 ± 10.7	62.3 ± 10.4	0.46
BMI (kg/m^2^)^b^	25.7 ± 5.0	25.5 ± 3.9	0.79
ASA classification^b^	2.1 ± 0.3	2.1 ± 0.4	0.53
Operated side (L/R) ^a^	26/34	31/27	0.27
PBV (L)^b^	3.5 ± 0.4	3.6 ± 0.5	0.19
Caprini scores^b^	5.1 ± 2.5	5.6 ± 2.6	0.46
Preoperative laboratory values			
Hemoglobin (g/L)^b^	130.5 ± 13.7	128.3 ± 13.2	0.36
Hematocrit (%)^b^	39.1 ± 3.7	38.7 ± 3.7	0.55
Platelet count (× 10^9^/L)^b^	182.7 ± 55.2	179.1 ± 45.5	0.73
Albumin (g/L)^b^	42.0 ± 6.1	43.0 ± 3.2	0.28
FDP (mg/L)^b^	3.1 ± 2.5	2.9 ± 2.4	0.70
D-Dimer (mg/L)^b^	1.0 ± 1.4	1.2 ± 1.4	0.46
PT (s)b	11.4 ± 0.7	11.3 ± 1.0	0.62
Surgical data			
Operative time (min)^b^	62.9 ± 13.1	63.6 ± 12.4	0.56
Preoperative knee function			
ROM (°)^b^	98.0 ± 22.4	96.6 ± 17.4	0.71
KSS clinical score^b^	35.8 ± 14.4	36.2 ± 15.8	0.89
KSS function score^b^	42.4 ± 11.6	41.2 ± 10.1	0.57
Knee circumference (cm)^b^	41.1 ± 4.0	41.4 ± 3.5	0.58
VAS pain score^b^	5.4 ± 1.4	5.8 ± 1.5	0.10

^a^The values are given as the number of patients

^b^The values are presented as the mean and the standard deviation

### Primary outcome

The total blood loss (primary outcome) was 671.7 ± 352.7 mL in Group A and 915.8 ± 243.4 mL in Group B, (*p* = 0.001), and there was a significant difference between the groups (Table 2).

**Table 2:** Primary and secondary outcomes regarding laboratory values and clinical outcomes after surgery

Variable	Group A	Group B	*P* value
Primary outcomes			
Total blood loss (mL)^b^	671.7 ± 352.7	915.8 ± 243.4	0.001
Secondary outcomes			
Postoperative transfusion			
Patients requiring transfusion	2 (3.33%)	2 (3.45%)	0.44
Units transfused (IU)	3.5	4	NA
Postop. laboratory values			
Hemoglobin (g/L)^b^			
POD 1	115.3 ± 13.9	117.1 ± 11.6	0.55
POD 3	106.0 ± 10.9	99.7 ± 8.4	0.001
POD 14	123.8 ± 10.3	121.3 ± 8.1	0.13
Hematocrit (%)^b^			
POD 1	35.1 ± 4.2	35.7 ± 3.2	0.43
POD 3	32.3 ± 2.9	30.0 ± 2.5	0.001
POD 14	37.0 ± 4.5	36.4 ± 4.6	0.45
FDP (mg/L)^b^			
POD 1	13.7 ± 7.8	24.9 ± 11.3	0.001
POD 3	6.9 ± 3.4	12.0 ± 10.0	0.001
POD 14	4.4 ± 2.5	5.2 ± 2.4	0.083
D-Dimer (mg/L)^b^			
POD 1	4.6 ± 2.2	8.4 ± 8.0	0.001
POD 3	1.5 ± 1.1	3.3 ± 2.1	0.001
POD 14	1.2 ± 0.9	1.3 ± 0.5	0.22
Postop. knee function			
ROM (°)^b^			
POD 1	99.7 ± 11.8	100.1 ± 11.7	0.58
POD 3	108.1 ± 4.9	107.4 ± 5.2	0.44
POD 14	110.9 ± 8.9	108.2 ± 9.4	0.11
PO 3 M	114.0 ± 7.0	113.7 ± 9.5	0.82
KSS clinical score^b^			
POD 3	48.2 ± 8.9	45.6 ± 8.5	0.32
POD 14	77.3 ± 6.4	75.2 ± 9.1	0.15
PO 3 M	82.8 ± 5.5	82.5 ± 4.3	0.73
KSS function score^b^			
POD 3	44.5 ± 10.6	43.1 ± 10.2	0.27
POD 14	68.6 ± 8.1	69.5 ± 8.4	0.53
PO 3 M	75.5 ± 5.6	76.7 ± 5.3	0.25
Knee circumference (cm)^b^			
POD 1	42.0 ± 3.7	43.8 ± 3.3	0.06
POD 3	43.1 ± 1.9	46.1 ± 3.7	0.001
POD 14	41.4 ± 2.9	44.9 ± 2.2	0.001
PO 3 M	41.1 ± 3.2	41.6 ± 3.0	0.28
VAS pain score^b^			
POD 1	3.8 ± 1.5	4.2 ± 2.0	0.26
POD 3	2.7 ± 0.8	2.9 ± 0.7	0.11
POD 14	2.2 ± 1.0	2.0 ± 0.8	0.32
PO 3 M	0.7 ± 0.9	0.6 ± 0.8	0.35
Hospital stay (day)^b^	3.7 ± 1.2	4.0 ± 0.9	0.15

### Secondary outcomes

#### Subcutaneous ecchymosis

During the period of follow-up, the morbidity of ecchymosis was higher in Group B (38 patients) than in Group A (7 patients), *P* = 0.001. The area of ecchymosis of the operative side limb in Group A was smaller than Group B (1.6% of the body surface area in group A vs 3.0% in group B, *P* = 0.001) (Table 2).

#### Postoperative laboratory values

No difference between the two groups was identified in Hb level or hematocrit on POD 1 or POD 14, but on POD 3, Hb and hematocrit in Group A were higher than in Group B (*P* = 0.001 and *P* = 0.001, respectively). The levels of FDP and D-Dimer were lower in Group A, and at the POD 1 (FDP: 13.7 vs 24.9, D-Dimmer:4.6 vs 8.4) and POD 3 (FDP: 6.9 vs 12.0, D-Dimmer:1.5 vs 3.3), there were significant differences (*P* < 0.05), but on POD 14, FDP and D-Dimmer were similar between the groups (Table 2).

#### Postoperative knee function and knee circumference

Knee ROM and KSS score were similar among the two groups during the 3-month follow-up. Similarly, the postoperative VAS was not significantly different between the groups at any follow-up point (Table 2).

The swelling of the lower limb in Group A was more mild than in Group B on POD 3 and POD 14. The circumference of the knee was 43.1 cm vs 46.1 cm (POD 3, *P* = 0.001) and 41.4 cm vs 44.9 cm (POD 14, *P* = 0.001) for Group A vs Group B, respectively but there was no difference between the two groups on POD 1 or the 3-month follow-up (Table 2).

#### Length of hospital stay and patient satisfaction

The length of hospital stay was 3.7 days in Group A VS 4.0 days in Group B, *P* = 0.15(Table 2). Patients in Group A had better satisfaction as there were 52 patients (86.7%) who were satisfied with the surgery, but only 46 patients (70.3%) in Group B were satisfied, but here was no difference between the two groups. The main reason that made patients feel uncomfortable were pain, swelling of the limb and ecchymosis (Table 3).

**Table 3:** Other outcomes including adverse events

Variable	Group A	Group B	P value
Ecchymoses morbidity	7	38	0.001
Area of ecchymoses(%)^b^	1.6 ± 0.7	3.0 ± 1.3	0.001
Symptomatic DVT	0	0	NA
Asymptomatic DVT	5	3	0.50
Superficial infection	0	0	1.00
Deep infection	0	0	NA
Hematoma	0	0	NA
Wound secretion	2	1	0.58
30-day mortality	0	0	NA
90-day readmission	0	0	NA
Satisfaction level			
Extremely satisfied	21	18	0.88
Very satisfied	19	17	
Somewhat satisfied	12	11	
Neither satisfied nor dissatisfied	7	10	
Somewhat dissatisfied	1	2	
Very dissatisfied	0	0	

#### Transfusion rate

Four patients received allogeneic blood transfusion due to a postoperative Hb of < 70 g/L and had symptoms indicative of anemia. Two were patients (3.3%, 3.5 U) in Group A and 2 (3.5%, 4.0 U) were in Group B, and there was no significant difference between the two groups (Table 2).

#### Complications

DVT morbidity, wound secretion and infection did not differ significantly between the two groups, and there were no mortality or readmission events during the follow-up period (Table 3).

## Discussion

Good blood conservation during the perioperative period can reduce blood loss and transfusion and decrease anemia, and patients who have higher levels of hemoglobin display better physical abilities and executive abilities during rehabilitation training [8, 30, 39–41]. Many studies including meta-analyses have proven that corrected anemia can reduce mortality after surgery [42–44]. Blood conservation strategies are one of the core components of the ERAS strategy for TKA, and TXA makes this strategy a reality.

TXA has been widely used to reduce blood loss and transfusion following TKA, especially in ERAS. Increasing numbers of studies have supported this fact [2, 15, 40, 43, 45–48]. In China, more than two hundred thousand primary TKAs are conducted each year. Considering the aging population and longer life expectancy, this number of TKAs will increase dramatically over time. TXA is useful for reducing swelling and inflammatory reactions after total joint arthroplasty [49, 50]. Although previous studies showed that hyperfibrinolysis continued for approximately 6–18 h after the operation, anticoagulants, such as low molecular heparin or rivaroxaban, might cause new bleeding. Fibrinolysis could persist at a low level, which is why some studies showed that prolonged use of TXA or multiple-dose drugs can further reduce blood loss and restrain postoperative fibrinolysis better than a single dose [23, 51–53]. We believe that prolonged use of TXA has more benefits for patients with total joint arthroplasty.

This study is the first study of long-term use of TXA after TKA. The results of our study show that, in the group with long-term use of TXA after the surgery (Group A), patients had lower total blood loss than Group B and patients in Group A had a higher hemoglobin than Group B on POD 3, that means when we prolonged the uses of TXA, it could reduce the blood lose from POD 1 to POD 3. The blood coagulation level as measured by fibrinolysis (D-Dimer) was lower in Group A than in Group B on POD 1 and POD 3. Patients in Group A had less ecchymosis morbidity, smaller ecchymosis area, and less swelling than Group B. Those results confirmed from another aspect that prolonged the use of tranexamic acid was helpful in inhibiting fibrinolysis and reducing the morbidity of swelling and subcutaneous ecchymosis of the lower limb after TKA. When we prolonged the use TXA after surgery, the total blood loss was comparable at two or three times the short-term use on the operation day and after the surgery [34, 54]. However, our scheme is more acceptable to patients because they did not need to take oral or intravenous TXA frequently on the operation day after general anesthesia.

The guidelines for the prevention of venous thromboembolism after orthopedic surgery in China suggest that, after total knee arthroplasty, we should use anticoagulants for at least 10 to 14 days [55, 56]. In the ERAS strategy, surgeons tried to find a balance of bleeding, hemostasis, anticoagulation and antiplasmin. In our institution, we attempt use TXA to achieve this balance. To date, our department has published dozens of papers and proved this conclusion [9, 14, 15, 26, 28, 30, 46, 54, 57]. Patients are discharged fewer than 3 days after total knee arthroplasty, but they should take oral anticoagulants, such as rivaroxaban, for more than 10 days at home to prevent VTE. There are few community health service institutions in China, and doctors there cannot supervise patient follow-up. When patients feel uncomfortable or anxious about the side effects of drugs, such as subcutaneous ecchymosis or swelling, they might travel hundreds of miles to meet with their surgeons. The results of our study proved that long-term, oral TXA after discharge from the hospital decreases ecchymosis and swelling caused by anticoagulants and made patients feel more comfortable and less anxious.

Although this study was carefully designed, there were still several limitations. First, the study population was small. For the incidence of ecchymosis and swelling, we believe that a larger sample size would be better powered to detect differences between the two groups, but the sample size calculations showed that our small sample was sufficient. Second, peak blood concentration occurs at approximately 3 h, and the half-life of oral TXA is approximately 2 h, but rivaroxaban reaches a peak 2 h after oral administration with a half-life of 4–6 h. Oral TXA might be not match the effect of rivaroxaban, and we plan to design another study and increase the frequency of oral TXA to obtain better results. Third, the 3-month follow-up might have concealed a different long-term safety profile for oral TXA. Forth, there were multiple secondary outcomes without correction that will be considered in future research. However, the half-life of TXA is short, and a 3-month follow-up period should have been adequate for observing attributable side effects or important early adverse reactions.

## Conclusion

This prospective, randomized, controlled trial of total knee arthroplasty using a fast-track protocol with no tourniquet and postoperative drain demonstrated that intravenous and subsequent long-term oral tranexamic acid provided less blood loss, less swelling, and less ecchymosis compared with short term TXA use without increasing the risk of complications.

## Data Availability

The datasets used and analyzed during the current study are available from the corresponding author on reasonable request.

## Abbreviations

ASA: The American Society of Anesthesiologists
ERAS: Enhanced recovery after surgery
FDP: Fibrin degradation products
IA: Intra-articularly
IV: Intravenous
KSS: Knee society score
PE: Pulmonary embolism
ROM: Range of motion
TKA: Total knee arthroplasty
TXA: Tranexamic acid
VTE: Venous thromboembolism
